# A Doherty Power Amplifier for Ultrasound Instrumentation

**DOI:** 10.3390/s23052406

**Published:** 2023-02-21

**Authors:** Hojong Choi

**Affiliations:** Department of Electronic Engineering, Gachon University, 1342 Seongnam-daero, Sujeong-gu, Seongnam 13120, Gyeonggi-do, Republic of Korea; hojongch@gachon.ac.kr

**Keywords:** Doherty power amplifier, ultrasound instrumentation, ultrasound transducer

## Abstract

The ultrasound instrumentation uses linear power amplifiers with low power efficiency, generating unwanted heat and resulting in the deterioration of the echo signal quality of measured targets. Therefore, this study aims to develop a power amplifier scheme to increase power efficiency while maintaining appropriate echo signal quality. In communication systems, the Doherty power amplifier has shown relatively good power efficiency while producing high signal distortion. The same design scheme cannot be directly applied to ultrasound instrumentation. Therefore, the Doherty power amplifier needs to be re-designed. To verify the feasibility of the instrumentation, a Doherty power amplifier was designed to obtain high power efficiency. The measured gain, output 1-dB compression point, and power-added efficiency of the designed Doherty power amplifier were 33.71 dB, 35.71 dB_m_, and 57.24% at 25 MHz, respectively. In addition, the performance of the developed amplifier was measured and tested using the ultrasound transducer through the pulse-echo responses. The output power with 25 MHz, 5-cycle, and 43.06 dB_m_ generated from the Doherty power amplifier was sent through the expander to the focused ultrasound transducer with 25 MHz and 0.5″ diameter. The detected signal was sent via a limiter. Afterwards, the signal was amplified by a 36.8 dB gain preamplifier, and then displayed in the oscilloscope. The measured peak-to-peak amplitude in the pulse-echo response with an ultrasound transducer was 0.9698 V. The data showed a comparable echo signal amplitude. Therefore, the designed Doherty power amplifier can improve the power efficiency used for medical ultrasound instrumentation.

## 1. Introduction

Ultrasound instrumentation is non-invasive compared to X-ray-based computed tomography and positron emission tomography [1,2,3,4]. As the semiconductor technology node is smaller, the fabrication cost per transistor is lower, while several features are integrated into wireless applications [5,6]. With this improvement, the size of ultrasound instrumentations can be compact, so it could be used in the emergency rooms and ambulances [7]. Therefore, several ultrasound companies have developed different types of ultrasound instrumentations with array-type transducers [8,9,10].

The typical ultrasound instrumentations are composed of ultrasound transmitters, receivers, and transducers [11,12,13]. Among the components in ultrasound instrumentations, the power amplifiers in the ultrasound transmitters and analog-digital-converters in the ultrasound receivers are the most critical electronic component units for direct current (DC) power consumption, respectively [14]. Therefore, the performances of the power amplifiers could affect the whole power consumptions of the ultrasound transmitters. Compared to general ultrasound instrumentations utilizing the alternating current (AC) power cords, portable medical ultrasound machines have limited battery lives [15,16]. Proper design and performances of the power amplifiers are complicated that can affect the performances of ultrasound instrumentations. Therefore, the power amplifiers for such instrumentations could be properly designed to obtain the adequate power efficiency of the ultrasound machines while generating improved performances, such as sensitivities and harmonic distortions of the echo signals generated by ultrasonic transducers [17]. For portable medical ultrasound instrumentations, power efficiency could be a more serious issue compared to general bench-top ultrasound instrumentations, because driving higher power in the portable ultrasound instrumentations can yield an adequate sensitivity of the ultrasound transducers, which are related to the performances of ultrasound instrumentations [18,19]. Therefore, high voltage driving powers are required here. However, they might lead to lower battery lives [20,21,22].

The general power amplifiers are classified into linear and non-linear power amplifiers used in ultrasound instrumentations [23,24]. The linear power amplifiers are Class-A-, Class-B-, and Class-AB-type power amplifiers with low power efficiency and low signal distortions [25]. For general ultrasound instrumentations using AC power cords, linear power amplifiers have been used because they provide low harmonic signal distortions generated by ultrasonic transducers that compensate for power efficiency characteristics [26].

In this paper, linear (Class-B and Class-AB) and non-linear (Class-D, Class-DE, and Class-E) power amplifiers are shown for general ultrasound imaging and high-intensity focused ultrasound (HIFU) applications. A Class-B power amplifier was developed for an ultrasound instrumentation [27]. A 0.03 MHz Class-B power amplifier was designed to produce an output voltage of 396 V [28]. A 10 MHz Class-B power amplifier with an output power of 3.09 W, an output voltage of 27.25 V, and an efficiency of 5.66% was designed [29]. A Class-AB power amplifier with an output voltage of 90 V and a bandwidth of 6.5 MHz was implemented [30]. An 18.5 MHz bandwidth Class-AB power amplifier was implemented [31]. A 50 MHz Class-AB power amplifier was designed [32].

The non-linear power amplifiers are Class-D-, Class-DE-, and Class-E-type power amplifiers with high power efficiency and high signal distortions [33,34]. A high voltage 3.5 kHz Class-D amplifier was designed [35]. A 0.1 MHz Class-D amplifier was designed [36]. A Class-DE power amplifier with a logic gate driver was developed [37]. A 1.54 MHz Class-E power amplifier was developed [38]. A Class-E inverter was designed to produce an optimal frequency of 40.07 kHz [39]. However, non-linear power amplifiers produce relatively high signal distortions.

To date, there is no Doherty power amplifier scheme developed for ultrasound applications. William H. Doherty first developed the Doherty power amplifier for radio communication system [40,41]. The Doherty power amplifier, which is one of the non-linear power amplifiers, has been used for low voltage communication systems with high power efficiency, but also high signal distortion. Higher power efficiency indicates that the power amplifier could consume a relatively lesser power consumption [42]. Therefore, the Doherty power amplifier could be one of the candidates for ultrasound instrumentation. However, one of the challenging technical issues is that the developed Doherty power amplifier must be properly working in the high voltage operations for ultrasound applications while still obtaining echo signal distortion of the ultrasound transducers. The design of such a power amplifier operating in high voltage operations is difficult to implement because the power transistor model in the large signal operations is less accurate than that in low voltage signal operations [43,44], even though other previous studies reported that the power transistor model is not accurate in low voltage signal operations. Compared to communication system applications, ultrasound applications must have adequate signal quality for harmonic ultrasound imaging applications [45,46,47]. Therefore, developed Doherty power amplifiers must be re-designed for ultrasound instrumentations. In addition, the selected components in the Doherty power amplifiers must properly work under high voltage operations.

This paper is organized as follows: Section 2 describes the schematic design, circuit analysis, and printed circuit board (PCB) implementation of the Doherty power amplifier for ultrasound instrumentation applications. Section 3 shows the experimental results of the Doherty power amplifiers, such as power efficiency and gain. In addition, the measured results are shown with ultrasound transducers because the Doherty power amplifier is customized for ultrasound applications. Section 4 presents the conclusion of the study.

## 2. Materials and Methods

**Figure 1:**
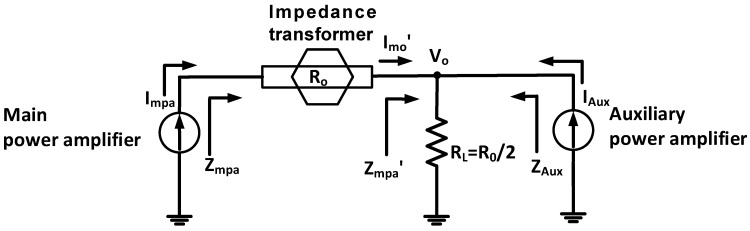
Equivalent circuit model of the Doherty power amplifier.

The Doherty power amplifiers operate according to the load modulation theory [43,48]. In other words, the main power amplifier works in lower input power ranges. The main and auxiliary power amplifiers work together in higher input power ranges to obtain relatively high efficiency in wide input power ranges. Figure 1 shows the equivalent circuit model of the Doherty power amplifier to explain the concept of the load modulation.

Using the Kirchhoff’s current law, the output voltage (V_o_) at the load impedance (R_L_) of the Doherty power amplifier can be expressed as follows [49,50,51]:

(1) $$V_{O}=\frac{R_{O}}{2}\left(I_{mo}\prime +I_{Aux}\right),$$

where I_mo_′ is the output current of the main power amplifier passing through the impedance transformer and I_AUX_ is the output current of the auxiliary power amplifier.

The gain (G) and output power (P_o_) of the Doherty power amplifier could be calculated as

(2) $$G=\frac{R_{O}}{2V_{i}}\left(I_{mo}\prime +I_{Aux}\right),\;P_{O}=V_{O}\left(I_{mo}\prime +I_{Aux}\right),$$

The load impedance at the main and auxiliary amplifiers (Z_mpa′_ and Z_AUX_) are expressed as

(3) $$Z_{mpa\prime }=\frac{V_{O}}{I_{mo}^{\prime }}=\frac{R_{O}}{2}\left(\frac{I_{mo}\prime +I_{Aux}}{I_{mo}\prime }\right),\left(0\leq I_{Aux}\leq I_{mo}^{\prime }\right),$$

(4) $$Z_{AUX}=\frac{V_{Out}}{I_{Aux}}=\frac{R_{O}}{2}\left(\frac{I_{mo}\prime +I_{Aux}}{I_{Aux}}\right).$$

The input impedance of the Doherty power amplifier can be calculated by the impedance transformer.

(5) $$Z_{mpa}=\frac{{R_{O}}^{2}}{Z_{mpa}\prime }=\frac{{2R}_{O}}{\left(1+\frac{I_{Aux}}{I_{mo}\prime }\right)},(0\leq \frac{I_{Aux}}{I_{mo}\prime }\leq 1)$$

The operating gate voltage of the auxiliary power amplifier is lower than that of the main amplifier. Therefore, the main power amplifier is only working for low input power ranges such that the impedance of the main power amplifier (Z_mpa_) is changed into the 2R_0_ [23]. The auxiliary power amplifier properly works such that the impedances of Z_mpa_′ and Z_mpa_ are closed to R_0_ as the input power increases and reaches to the maximum input powers [23].

**Figure 2:**
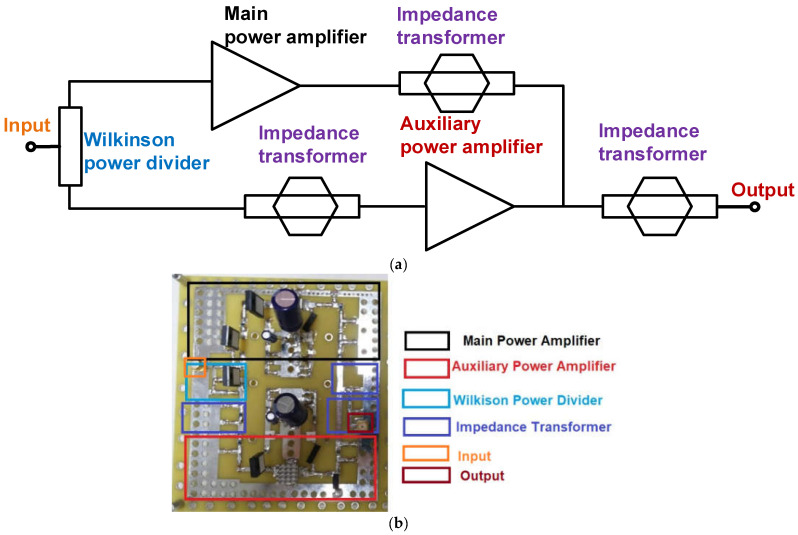
(**a**) Configuration and (**b**) implemented PCB of the developed Doherty power amplifier.

Figure 2a shows the block diagram of the Doherty power amplifier. The Doherty power amplifier consists of the main and auxiliary power amplifiers with impedance transformers. Figure 2b shows the implemented main, auxiliary, and developed Doherty power amplifiers on the custom-made PCB. For the power amplifier to work properly under high voltage operations, selected discrete components are high voltage or high current tolerant. The choke inductors with a maximum current of 2A and the transistors with a maximum drain-source voltage of 65 V were selected. In addition, the square heat-sink was attached on top of the transistors to reduce the temperature effects. In the output port, the high power resistors with maximum voltage of 250 V were used. The electrolytic capacitors with maximum voltage of 50 V in the gate and drain side of the transistor were also used. The next paragraph shows the detail architecture and operating mechanisms of the designed Doherty power amplifier.

**Figure 3:**
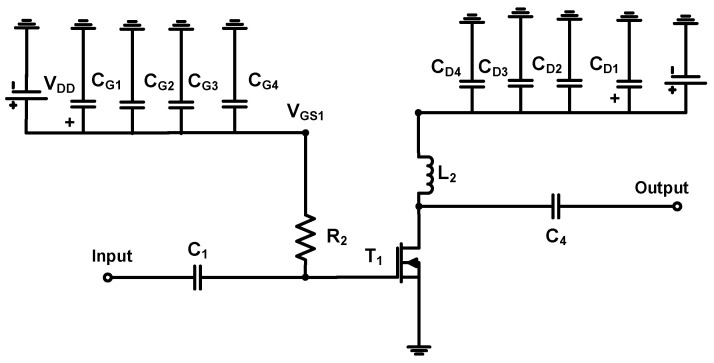
Schematic of the main power amplifier.

Figure 3 shows the schematic of the main power amplifier. The main transistor in power amplifiers is a lateral diffusion metal oxide semiconductor (LDMOS) transistor, which is suitable for high voltage operation because of considerably high breakdown voltage [52]. The transistor (T_1_) was used for main and auxiliary power amplifiers. The input and output DC coupling capacitors (C_1_ and C_4_) were used in the main power amplifier to block unwanted DC voltages [53]. The bias feed resistor (R_2_) was used to provide the bias voltage of the transistor (T_1_). The choke inductor (L_2_) was used to minimize the supply voltage reduction [54,55]. According to the transistor datasheet, the same values of the electrolytic capacitors and ceramic capacitors were used. The electrolytic capacitor (C_G1_ = 10 μF) and ceramic capacitors (C_G2_, C_G3_, and C_G4_ = 0.1 μF, 1000 pF, and 47 pF, respectively) in the gate side of the transistor (T_1_) and the electrolytic capacitor (C_D1_ = 220 μF) and ceramic capacitors (C_D2_, C_D3_, and C_D4_ = 220 μF, 0.1 μF, 100 pF, and 47 pF, respectively) in the drain side of the transistor (T_1_) were used. In the input port, low pass filters were composed of the resistor (R_1_), inductor (L_1_), and capacitor (C_1_). In the output port, low pass filters composed of the capacitors (C_4_, C_5_, and C_6_) and inductors (L_3_ and L_4_) were used.

**Figure 4:**
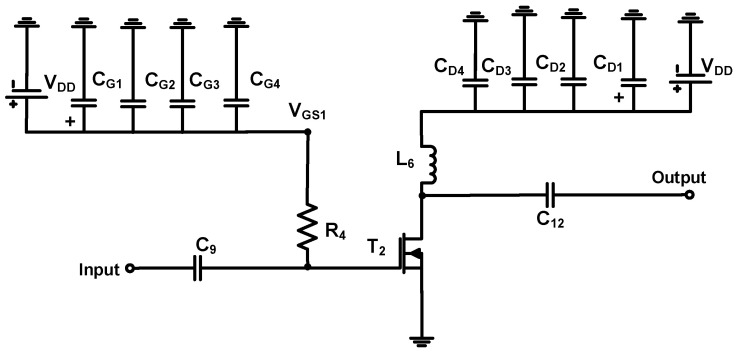
Schematic of the auxiliary power amplifier.

The schematic of the auxiliary power amplifier is shown in Figure 4. The input and output DC coupling capacitors (C_9_ and C_12_) were used in the auxiliary power amplifier to block unwanted DC voltages [56]. The bias feed resistor (R_4_) was used to provide the bias voltages to the transistor (T_2_). The choke inductor (L_6_) was used to minimize the voltage reduction [57,58]. The electrolytic capacitor (C_G1_ = 10 μF) and ceramic capacitors (C_G2_, C_G3_, and C_G4_ = 0.1 μF, 1000 pF, and 47 pF, respectively) for the gate side in the transistor (T_2_) and the electrolytic capacitor (C_D1_ = 220 μF) and ceramic capacitors (C_D2_, C_D3_, and C_D4_ = 0.1 μF, 100 pF, and 47 pF, respectively) for the drain side in the transistor (T_2_) were used. In the input port, low pass filters were composed of the capacitors (C_7_ and C_8_) and inductor (L_4_). In the output port, low pass filters composed of the resistor (R_5_), capacitors (C_13_ and C_14_), and inductor (L_7_) were used.

For the Doherty power amplifier used in communication applications, the impedance transformer is implemented as transmission lines [59,60]. However, the working frequencies and voltages in ultrasound systems are less than a few hundred MHz and are higher than a few volts, which are high voltage ranges such that the impedance transformer was re-designed used for ultrasound applications. The developed impedance transformers were only used [33]. The input signals were spitted equally using the Wilkinson power divider circuit [41]. Subsequently, they were transmitted to the main and auxiliary amplifiers.

The impedance transformer was implemented to remove the harmonic components. The Wilkinson power divider was used to equally divide the input. The Wilkinson power divider was composed of three capacitors, two inductors, and one resistor (R_6_).

The simulated results of the Doherty power amplifier under high voltage operations were not provided. First, the simulation results of the signal distortions of the power amplifiers do not provide accurate results as mentioned in a previous study [34]. The simulation libraries of some discrete components, such as power resistors and choke inductors, which are composed of the Wilkinson power divider, as well as the impedance transformer, are not provided from the manufacturers. In addition, the designed Doherty power amplifier must work under high voltage operations such that the simulated results do not show the accurate results because the real performance of the power amplifier is affected by the temperature effect [61]. Therefore, some discrepancy values between expected and measured results of the Doherty power amplifier performance are expected.

## 3. Results and Discussion

### 3.1. Performance Measurements for Main, Auxiliary, and Doherty Power Amplifiers

One of the important specifications of the power amplifiers is the output power versus the input power with the output 1-dB compression point (OP_1dB_), gain, and power-added efficiency (PAE). The OP_1dB_ is the index of the linearity of the power amplifier [62]. The linearity is related with the signal distortions of the amplifier [63]. The PAE is the index of the power consumption of the power amplifier and is defined as the ratio of the AC output power to the DC input power [64].

**Figure 5:**
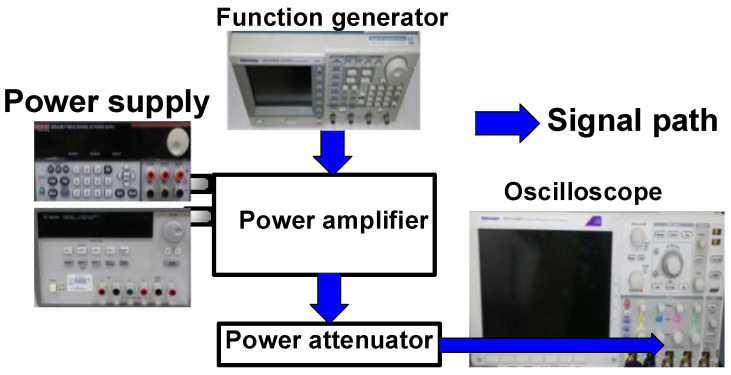
Measurement setup for output power, gain, P_1dB_, and PAE of the main, auxiliary, and Doherty power amplifiers.

Two power supplies (E3631A and 2231A-3-30, Keysight Technology, Santa Rosa, CA, USA) provided DC to the power amplifiers. For obtaining the output power and PAE, the five-cycle pulses generated from function generator (AFG3252C, Tektronix Inc., Beaverton, OR, USA) were utilized for the ultrasound applications because multi-cycle pulses were applied to operate the ultrasound transducers [65]. These pulses were amplified using power amplifiers, and their amplified pulses were displayed on the oscilloscope through a power attenuator. Figure 5 shows the experimental setup to measure the output power and PAE.

**Figure 6:**
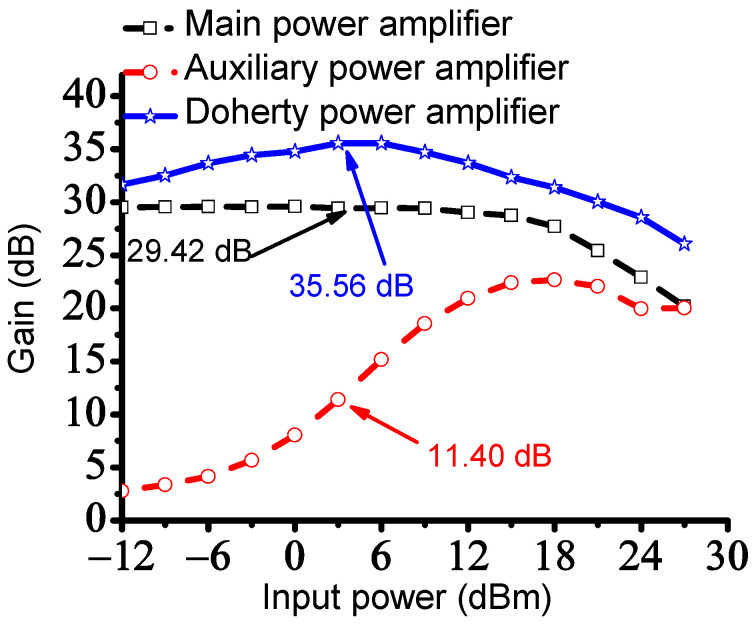
Measured gain versus input power of the main, auxiliary, and Doherty power amplifiers.

Figure 6 shows the measured gain of the main, auxiliary, and Doherty power amplifiers. The gain of the Doherty power amplifier was improved with the help of the main and auxiliary power amplifiers. The input power was calculated by multiplying the measured voltage and current signals. The operating gate voltage of the auxiliary power amplifier is lower than that of the main power amplifier. Therefore, the gain of the auxiliary power amplifier (2.78 dB) was lower than that of the main power amplifier (29.51 dB) at input power of −12 dB_m,_ as shown in Table 1. As described in Equation (4), the impedance of the auxiliary power amplifier is closed to R_0_ as the input current (I_AUX_) increases. Therefore, the auxiliary power amplifier properly works such that the gain of the Doherty power amplifier was saturated at a certain input power level. The gain of the main, auxiliary, and Doherty power amplifiers were measured as 29.42, 11.40, and 35.56 dB at 3.0 dB_m_, respectively, and the gain of the main, auxiliary, and Doherty power amplifiers were measured as 29.04, 20.95, and 33.71 dB at 12 dB_m_, respectively.

Table 1 shows the measured gain versus the input power performance of the main, auxiliary, and Doherty power amplifiers.

The Doherty power amplifier is one of the non-linear power amplifiers with good PAE at relatively high signal distortions [41]. However, ultrasound echo signal distortions could affect the image resolution [66]. Therefore, the designed Doherty power amplifier for ultrasound applications must have good PAE and adequate signal distortions. This indicates that the performance trade-off between the PAE and signal distortions could be compromised if we designed the Doherty power amplifier used for ultrasound transducers.

**Figure 7:**
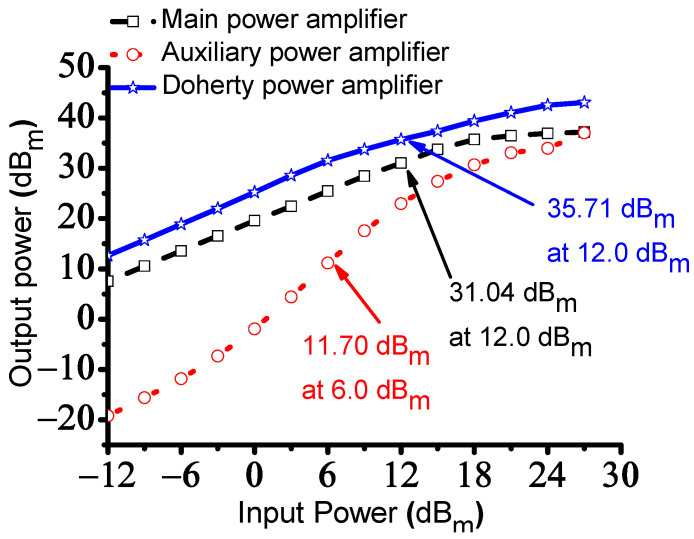
Measured output power versus input power with OP_1dB_ of the main, auxiliary, and Doherty power amplifiers.

**Figure 8:**
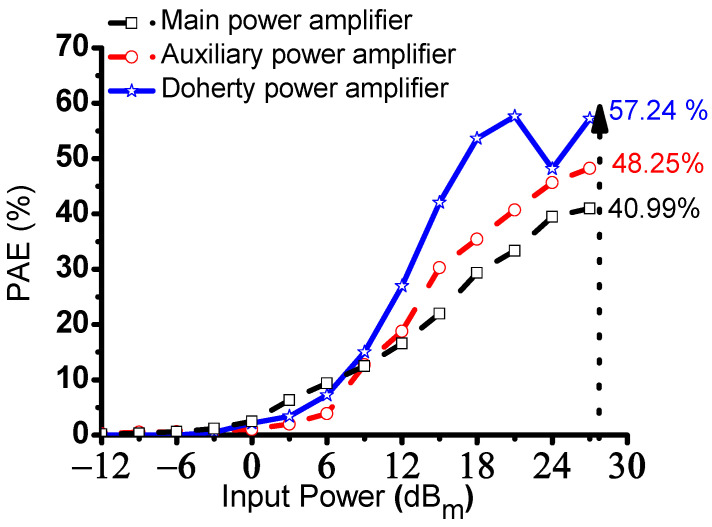
Measured PAE versus input power of the main, auxiliary, and Doherty power amplifiers.

Figure 7 shows the measured output power versus input power with OP_1dB_ of the main, auxiliary, and Doherty power amplifiers. The OP_1dB_ of the main, auxiliary, and Doherty power amplifiers were measured as 31.04 dB_m_ at 12.0 dB_m_, 11.70 dB_m_ at 6.0 dB_m_, and 35.71 dB_m_ at 12.0 dB_m_ input powers, respectively, as shown in Figure 8. As mentioned before, the gate voltage of the auxiliary power amplifier is lower than that of the main power amplifier so the output power of the auxiliary power amplifier (−19.22 dB_m_) was lower than that of the main power amplifier (7.51 dB_m_) at an input power of −12 dB_m_ as shown in Table 2. However, the impedances of the main and auxiliary power amplifiers were reached to R_0_ so that the output power of both power amplifiers would be increased accordingly. In the figure, the measured OP_1dB_ (35.71 dB_m_) of the Doherty power amplifier was improved compared to those (11.70 dB_m_ at 31.04 dB_m_) of the main and auxiliary power amplifier accordingly.

Table 2 lists the measured output power versus the input power performance of the main, auxiliary, and Doherty power amplifiers.

Figure 8 shows the measured PAE of the main, auxiliary, and Doherty power amplifiers. Because of the load modulation condition, the impedances of the main power amplifier and Doherty power amplifier (Z_mpa_′ and Z_mpa_) in the maximum power level are close to R_0_ [41]. Even though the input power increases, the impedances of the main and auxiliary power amplifiers were adjusted, so substantial output powers of the main and auxiliary power amplifiers were fed into the output power of the Doherty power amplifier [41]. Therefore, the PAE of the Doherty power amplifier would be increased and then reach the maximum PAE at the saturation point. In this figure, the measured PAE of the main, auxiliary, and Doherty power amplifiers were 40.99%, 48.25%, and 57.24% at an input power of 27.0 dB_m_, respectively. The measured PAE of the Doherty power amplifiers shows good PAE values for portable instrumentation.

Table 3 summarizes the measured PAE versus the input power of the main, auxiliary, and Doherty power amplifiers. These measurement results confirm that the Doherty power amplifier could be useful for ultrasound instrumentation.

### 3.2. Pulse-Echo Measurement with Ultrasound Transducer

The Doherty power amplifier can optimize power consumption such that good power efficiency could be achieved. However, the harmonic signal distortions of the echo signals are also important in obtaining high signal quality in ultrasound instrumentation [67,68,69]. Therefore, the performances of the power amplifiers were measured in the pulse-echo measurement setup, which is a common evaluation method of ultrasound devices [70].

**Figure 9:**
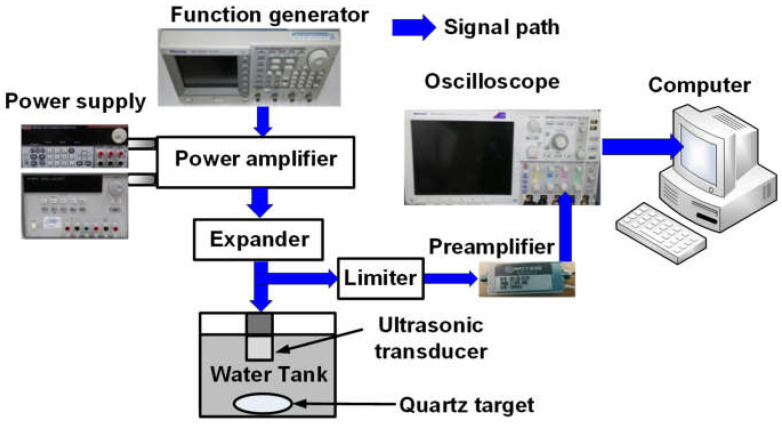
Pulse-echo measurement setup using power amplifiers with an ultrasound transducer.

Figure 9 illustrates the pulse-echo measurement when using the main, auxiliary power amplifiers, and Doherty power amplifiers. The DC voltages generated by two power supplies (E3531A and 2231A-3-30) and five-cycle pulse signals fed by the function generator (AFG3252C) were applied to the power amplifier. The high voltage pulse signals amplified by the power amplifier fed into a 25 MHz and 0.5″ diameter focused ultrasound transducer through an expander. The expander consists of a cross-coupled diode pair in the aluminum enclosure box. The echo signals detected by the ultrasound transducer pass through a limiter. Then, they were amplified by a preamplifier with a 36.8 dB voltage gain. The limiter is composed of a 50-ohm resistor shunt with a cross-coupled diode in the aluminum enclosure box. Finally, these echo signals obtained in the oscilloscope were recorded in the computer.

**Figure 10:**
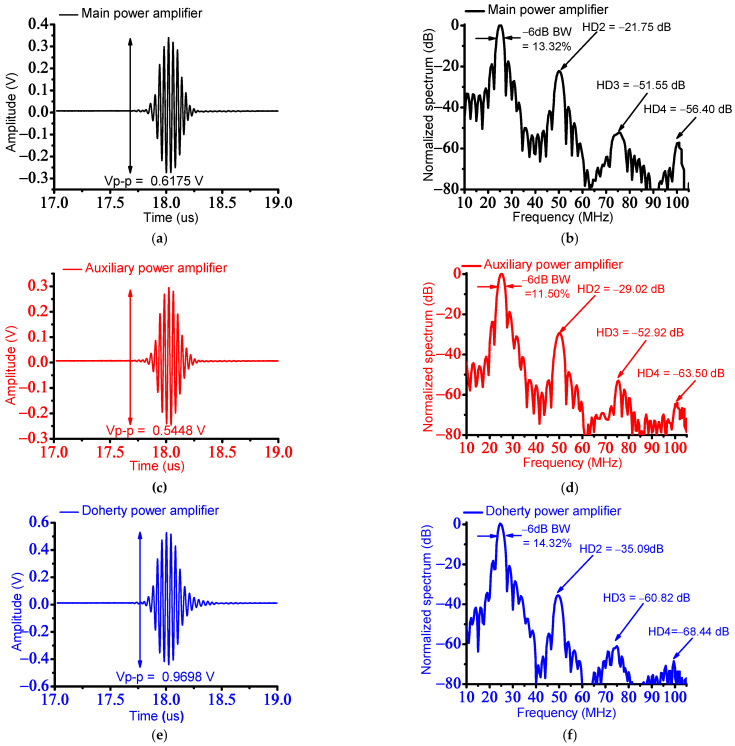
Amplitudes of echo signals and normalized spectrum from a 25 MHz ultrasound transducer. (**a**) The amplitudes and (**b**) spectrum of the echo signal when using the main power amplifier. (**c**) The amplitudes and (**d**) spectrum of the echo signal when using the auxiliary power amplifier. (**e**) The amplitudes and (**f**) spectrum of the echo signal when using Doherty power amplifier.

Figure 10 shows the data of the amplitudes and spectra when using a 25 MHz ultrasound transducer with a power amplifier. The outputs of the main and auxiliary power amplifiers would be properly increased, so the Doherty power amplifier would be effectively improved; therefore, the measured echo signal amplitudes of the main, auxiliary, and Doherty power amplifiers were shown. With the trial and error method, the high PAE and relatively adequate harmonic distortion performances of the designed Doherty power amplifier were obtained because those performance parameters of the Doherty power amplifier have a trade-off relationship.

As shown in Figure 10a,c,e, the measured amplitudes of the echo signals when using the main, auxiliary, and Doherty power amplifiers were 0.6175 V_p-p_, 0.5448 V_p-p_, and 0.9698 V_p-p_, respectively, because the Doherty power amplifier has the highest gain among others. As shown in Figure 10b,d,f, the measured −6 dB bandwidths of the main, auxiliary, and Doherty power amplifiers were 13.32%, 11.50%, and 14.32%, respectively. The measured second, third, and fourth harmonic-distortion components (HD2 = −35.09 dB, HD3 = −60.82 dB, and HD4 = −68.44 dB) of the echo signals generated with the Doherty power amplifier were lower than those of the measured second, third, and fourth harmonic-distortion components (HD2 = −21.75 dB, HD3 = −51.55 dB, and HD4 = −56.40 dB) of the echo signals generated by the main power amplifier, respectively. The calculated total harmonic distortions (THDs) of the echo signals of the ultrasound transducer driven by the main, auxiliary, and Doherty power amplifiers were −43.48 dB, −58.00 dB, and −70.15 dB, respectively.

Table 4 summarizes measured performance of the peak-to-peak amplitude, −6 dB bandwidths, and HD2, HD3, HD4, and THD of the main, auxiliary, and Doherty power amplifiers. The measured performance of the Doherty power amplifier is improved, as shown in Table 4.

Table 5 summarizes the comparison of the currently developed power amplifiers for ultrasound transducer applications. The developed Doherty power amplifier has high PAE because the power amplifier performance decreased because of the non-linear characteristics of the transistor [71]. The efficiency of the power amplifier can be obtained by the output power divided by input DC power so the efficiency of the Doherty power amplifier was added in Table 5. A 325LA commercial amplifier has harmonic level less than −23 dB_c_. Each power amplifier scheme has different target parameters, such as output power, HD3, or efficiency, because there could be trade-off between each parameter. Therefore, each power amplifier has a higher performance parameter, scarifying other performance parameters.

## 4. Conclusions

The performances of the ultrasound instrumentations are restricted by the limited power consumption because of the limited battery life for severe environments in emergency rooms and ambulances. Due to limited power restriction, the structure size and numbers of array transducers, which are components in the ultrasound instrumentations, are smaller. Therefore, this can be the bottleneck of the echo signal performances and view angles of the target for ultrasound instrumentations.

The power amplifier used in the ultrasound instrumentations is one of the problems for consuming power and generating unwanted heat, thus reducing the performances of the instrumentation. A new type of Doherty power amplifier was developed to reduce DC power consumption of the power amplifier while producing adequate sensitivities of the ultrasound transducers. For communication areas, the Doherty power amplifier has been used for communication applications. However, this scheme has never been used for any ultrasound transducer applications and cannot be directly used because of different operation mechanisms and environments. Therefore, the new Doherty power amplifier scheme must be developed to be applied for ultrasonic transducers. The Doherty power amplifier was developed with the main and auxiliary power amplifier, impedance transformer, and Wilkinson power divider. The gain and OP_1dB_ of the Doherty power amplifier were measured as 33.71 dB and 35.71 dB_m_ at 12 dB_m_, respectively. The measured PAE of the Doherty power amplifier was 57.24% at 27.0 dB_m_.

To verify the feasibility, the pulse-echo responses were performed, and the performance of the power amplifier was measured using a 25 MHz ultrasound transducer. In the pulse-echo response with the ultrasound transducer, the measured peak-to-peak voltage amplitude of the echo signal when using the Doherty power amplifiers was 0.9698 V_p-p_. The measured −6 dB bandwidth when using Doherty power amplifiers was 14.32%. The measured second, third, and fourth harmonic-distortion components of the signals generated with the Doherty power amplifier are HD2 = −35.09 dB, HD3 = −60.82 dB, and HD4 = −68.44 dB, respectively. The measured THD was −70.15 dB. These measurement data show adequate harmonic distortions of the echo signals of the ultrasound transducers while consuming the low DC power consumption of the power amplifier. Therefore, the newly developed Doherty power amplifier can potentially be used for maintaining the performances of medical ultrasound instrumentation by improving the power-added efficiency of the power amplifier.

## Figures and Tables

**Table 1:** Measured gain versus input power of the main, auxiliary and Doherty power amplifiers.

Input Power (dB_m_)	Gain (dB)		
	Main Power Amplifier	Auxiliary Power Amplifier	Doherty Power Amplifier
−12	29.51	2.78	31.66
−9	29.56	3.36	32.52
−6	29.57	4.14	33.69
−3	29.56	5.67	34.45
0	29.60	8.05	34.78
+3	29.42	11.40	35.56
+6	29.45	15.17	35.56
+9	29.44	18.56	34.72
+12	29.04	20.95	33.71
+15	28.74	22.40	32.36
+18	27.71	22.69	31.39
+21	25.44	22.08	30.04
+24	22.92	19.95	28.57
+27	20.21	20.04	26.06

**Table 2:** Measured output power versus input power of the main, auxiliary, and Doherty power amplifiers.

Input Power (dB_m_)	Output Power (dB_m_)		
	Main Power Amplifier	Auxiliary Power Amplifier	Doherty Power Amplifier
−12	7.51	−19.22	12.66
−9	10.56	−15.64	15.72
−6	13.57	−11.86	18.89
−3	16.56	−7.33	21.99
0	19.60	−1.950	25.22
+3	22.42	4.400	28.56
+6	25.45	11.70	31.56
+9	28.44	17.56	33.72
+12	31.04	22.95	35.71
+15	33.74	27.40	37.36
+18	35.71	30.69	39.39
+21	36.44	33.08	41.04
+24	36.92	33.95	42.57
+27	37.21	37.04	43.06

**Table 3:** Measured PAE versus input power of the main, auxiliary, and Doherty power amplifiers.

Input Power (dB_m_)	Main Power Amplifier	PAE (%)	
		Auxiliary Power Amplifier	Doherty Power Amplifier
−12	0.26	0.17	0.0011
−9	0.52	0.32	0.0015
−6	0.61	0.61	0.01
−3	0.81	1.18	0.54
0	1.01	2.49	2.15
+3	2.02	6.33	3.41
+6	3.89	9.38	7.24
+9	12.72	12.46	15.00
+12	18.79	16.55	26.97
+15	30.26	21.98	42.04
+18	35.45	29.32	53.62
+21	40.72	33.33	57.64
+24	45.66	39.48	48.16
+27	48.25	40.99	57.24

**Table 4:** Summary of the measured performance of the main, auxiliary, and Doherty power amplifiers with an ultrasound transducer.

	Amplitude (V_p-p_)	−6 dB BW (%)	HD2 (dB)	HD3 (dB)	HD4 (dB)	THD (dB)
Main Power Amplifier	0.6175	13.32	−21.75	−51.55	−56.40	−43.48
Auxiliary Power Amplifier	0.5448	11.50	−29.02	−52.92	−63.50	−58.00
Doherty Power Amplifier	0.9668	14.32	−35.09	−60.82	−68.44	−70.15

**Table 5:** Summary of the linear and non-linear power amplifiers for ultrasound applications.

	Circuit Architecture	Output Power (dB_m_)	Output Power (W)	Output Voltage (V)	Center Frequency or −3 dB BW (MHz)	Gain (dB)	HD2 (dB)	HD3 (dB)	HD4 (dB)	THD (dB)	PAE (%)	Efficiency (%)	Applications
[28]	Class-B		1120	396	0.03								Parametric Transducer
[29]	Class-B		3.09	27.25	10							5.66	Piezoelectric Actuator
[30]	Class-B			90	6.5								Piezoelectric Transducer
[31]	Class-AB			31.5	18.5								Ultrasound Imaging
[32]	Class-AB			200	50								Ultrasound Imaging
[35]	Class-D			125	0.035	43.5							Dielectric Transducer
[36]	Class-D				0.1							95	Piezoelectric Load
[37]	Class-DE		0.8	20	1.1			−16.4					HIFU Therapy
[38]	Class-E			100	1.54								High Power Driver
[39]	Class-E		0.219		0.04								Piezoelectric Transducer
This Work	Doherty	35.71			25	33.71	−35.09	−60.82	−68.44	−70.15	57.24		Piezoelectric Transducer

## Data Availability

The data presented in this study are included within the article.
